# Evaluating the Interacting Influences of Pollination, Seed Predation, Invasive Species and Isolation on Reproductive Success in a Threatened Alpine Plant

**DOI:** 10.1371/journal.pone.0088948

**Published:** 2014-02-14

**Authors:** Paul D. Krushelnycky

**Affiliations:** Department of Plant and Environmental Protection Sciences, University of Hawai’i at Mānoa, Honolulu, Hawai’i, United States of America; Institut Mediterrani d’Estudis Avançats (CSIC/UIB), Spain

## Abstract

Reproduction in rare plants may be influenced and limited by a complex combination of factors. External threats such as invasive species and landscape characteristics such as isolation may impinge on both pollination and seed predation dynamics, which in turn can strongly affect reproduction. I assessed how patterns in floral visitation, seed predation, invasive ant presence, and plant isolation influenced one another and ultimately affected viable seed production in Haleakalā silverswords (*Argyroxiphium sandwicense* subsp. *macrocephalum*) of Hawai’i. Floral visitation was dominated by endemic *Hylaeus* bees, and patterns of visitation were influenced by floral display size and number of plants clustered together, but not by floral herbivory or nearest flowering neighbor distance. There was also some indication that Argentine ant presence impacted floral visitation, but contradictory evidence and limitations of the study design make this result uncertain. Degree of seed predation was associated only with plant isolation, with the two main herbivores partitioning resources such that one preferentially attacked isolated plants while the other attacked clumped plants; total seed predation was greater in more isolated plants. Net viable seed production was highly variable among individuals (0–55% seed set), and was affected mainly by nearest neighbor distance, apparently owing to low cross-pollination among plants separated by even short distances (>10–20 m). This isolation effect dominated net seed set, with no apparent influence from floral visitation rates, percent seed predation, or invasive ant presence. The measured steep decline in seed set with isolation distance may not be typical of the entire silversword range, and may indicate that pollinators in addition to *Hylaeus* bees could be important for greater gene flow. Management aimed at maintaining or maximizing silversword reproduction should focus on the spatial context of field populations and outplanting efforts, as well as on conserving the widest possible range of pollinator taxa.

## Introduction

Threatened and endangered plant species often suffer from a complex host of threats that may impinge on various stages of their life histories [1], [2]. This includes factors that limit fecundity [3], hence understanding the reproductive ecology of threatened or rare plants can be crucial for planning mitigation and recovery strategies. Such knowledge may be particularly important for species that are self-incompatible, and thus reliant on pollinators for successful reproduction, because they will be sensitive to forces that impact their pollinators in addition to those that affect them directly [4].

Reproductive success in plants is influenced by their intrinsic characteristics, the animals that interact with them, and the spatial context of the community in which they are embedded [5]–[8]. This applies both to pollination and seed predation dynamics, two key determinants of viable seed output. Thus, the identity, number and behavior of floral visitors affects pollination rates [9]–[13], and different seed predators may reduce seed production or survival to different degrees [14]. Furthermore, it has been demonstrated repeatedly that spatial factors such as plant isolation, population size or density, and degree of population fragmentation may strongly influence the number and composition of floral visitors [15]–[17], amount of pollination [18]–[21], and intensity of seed predation [22]–[24]. In addition, pollinators and seed predators may respond differently to each of these spatial attributes, and both may be impacted by invasive species or other stressors [25]–[28]. Although understanding the nature of each of these dynamics in isolation may often be of intrinsic interest, it is also evident that taking each into account becomes necessary when evaluating the importance of any single factor, and when assessing overall patterns in net seed production.

Comprehensive assessments of the various interacting forces impinging on plant reproduction are relatively uncommon, particularly for threatened or rare species. In this study, I investigated multiple factors that may limit seed production in a locally endemic tropical alpine plant in Hawai’i the Haleakalā silversword (*Argyroxiphium sandwicense* subsp. *macrocephalum* (Gray) Meyrat). The Haleakalā silversword, or ‘āhinahina, is a long-lived, large (up to 1 m diameter), monocarpic rosette plant in the Asteraceae family, that takes its name from its long slender leaves that are densely covered with silvery pubescence [29]. It is restricted to the top of Haleakalā volcano on the island of Maui, and suffered large population declines in the early part of the 20th century, but subsequently rebounded as a result of protection and management (see Methods and Materials). It is now a key biological component in its relatively barren cinder alpine habitat [30], and despite the fact that it currently numbers in the tens of thousands, has been given threatened status under the U.S. Endangered Species Act because of its restricted range, relatively recent recovery, and continued vulnerability to threats from invasive species [31].

The latter threat refers primarily to the Argentine ant, *Linepithema humile*, a highly destructive invasive species that greatly reduces native arthropod diversity and abundance [32], [33], and which has begun to spread into the silversword’s range. Because this ant was previously tied to large declines in nesting density of endemic yellow-faced bees (*Hylaeus* spp., family Colletidae) at Haleakalā, it was hypothesized that it could indirectly impact silversword reproduction by suppressing this key pollinator group [25]. Multiple species of these solitary bees, which belong to the only genus of native bees in Hawai’i, nest primarily or exclusively in the ground at this alpine site [34], and this likely increases their vulnerability to predation or displacement by Argentine ants. Anecdotal observations identify *Hylaeus* bees as the main pollinators for Haleakalā silverswords [35], [36], lending credibility to the proposed mechanism of reproductive impact, but to date pollination dynamics have not been quantified.

Earlier concerns regarding silversword population viability focused heavily on pre-dispersal destruction of seeds by native insect herbivores [37], [38], but also without much quantitative evidence. Two endemic species are responsible for nearly all of this highly visible damage: a moth (*Rhynchephestia rhabdotis*, Lepidoptera: Pyralidae) whose caterpillars feed in the inflorescence stems and flower heads, and a fruit fly (*Trupanea cratericola*, Diptera: Tephritidae) whose larvae feed in the flower heads. This herbivory could reduce reproductive output not only through seed predation but also by influencing pollination rates, if pollinator behavior is affected by feeding damage in flower heads. Interestingly, Argentine ants could have a positive indirect effect on silversword reproduction by reducing populations of these herbivores or interfering with their oviposition [39], [40], in contrast to their hypothesized effect involving pollinators.

Finally, spatial context is likely to be extremely important for Haleakalā silversword reproduction, as this plant is semelparous and strongly self-incompatible [36], [41]. Hence, degree of isolation could greatly influence the amount of successful cross-pollination and lifetime fecundity realized by individuals. This is potentially amplified by the clumped nature of the silversword population, characterized by numerous aggregations often separated by large areas devoid of plants [42]. Superimposed on this spatial pattern is a strong temporal fluctuation in annual flowering [43], which results in widely different effective population sizes from year to year.

A reproductive Allee effect has in fact been documented for Haleakalā silverswords, with a strong positive association between annual number of flowering plants and mean percent seed set [36], presumably due to greater spatial isolation of flowering individuals in years with fewer total flowering plants. An explicit negative relationship between seed set and distance to the nearest flowering neighbor was measured at one site for the Haleakalā silversword’s sister subspecies, the Mauna Kea silversword (*Argyroxiphium sandwicense* DC subsp. *sandwicense*) [44], but has not yet been characterized for silverswords at Haleakalā. Not only is this an important relationship to quantify in its own right, but it may also be crucial to account for when evaluating the roles of the other factors discussed above. Moreover, individual-based measures of isolation may often be better predictors of Allee effects on reproduction than measures of population size [45]. This condition is likely to be true for Haleakalā silverswords, where the number and spatial configuration of flowering plants can vary greatly across the landscape as well as between years, and can result in a disconnect between local inter-plant distances and population-wide flowering totals.

Haleakalā silverswords therefore exemplify the complex set of forces that may influence reproduction in rare and threatened plant species. I took advantage of natural variation across a landscape to evaluate simultaneously the importance of patterns in floral visitation, seed predation, invasive ant presence, and plant isolation for silversword seed production, and to determine how each of these factors may influence the others. Understanding these relationships is central to developing any management plans that might be needed, and should be informative for rare plant populations and species elsewhere.

## Materials and Methods

### Ethics Statement

Research, access and collection permits for native invertebrates and silversword seeds were issued by the National Park Service and the U.S. Fish and Wildlife Service.

### Study Site and Organism

Unspecified references to silversword plants in this study refer to the Haleakalā subspecies, *A. sandwicense macrocephalum*. Its sister taxon, the Mauna Kea silversword (*A. s. sandwicense*), is a federally listed Endangered subspecies restricted to the top of Mauna Kea volcano, Hawai’i Island. Haleakalā silverswords grow on the often barren cinder cones, cinder flats and rocky cliffs of Haleakalā crater and crater rim, in the alpine zone from 2150 to 3050 m elevation, almost exclusively within Haleakalā National Park.

The total Haleakalā silversword population has varied considerably in abundance over time. It reached an estimated low of perhaps 4000 plants in the 1920s–1930s owing to ungulate browsing and human vandalism, subsequently recovered strongly due to management actions, with peak numbers estimated at roughly 65,000 plants in 1991, and more recently began declining again as a result of warmer and drier climate conditions [31], [42], [43]. From June to September, a highly variable number of mature plants flower and then die (from 0 to a recorded high of 6632 in 1991), each producing a capitulescence (a large stalked inflorescence) containing dozens to 300+ capitula (flowerheads), which themselves contain dozens to 600+ hermaphroditic florets. Pistils are regularly exposed to self-pollen as a result of recurvature of the two stigmatic lobes onto anther surfaces, but this only rarely results in fertilization [41]. Self-incompatibility is thought to be controlled by a multi-allelic sporophytic incompatibility system [41], [44], and out-crossing with compatible individuals is needed to attain seed set exceeding approximately 0.5–1% [36], [41]. Fertilized florets produce filled achenes (fruits) containing a single viable seed, while unfertilized florets produce empty achenes; filled and unfilled achenes are outwardly indistinguishable.

**Figure 1:**
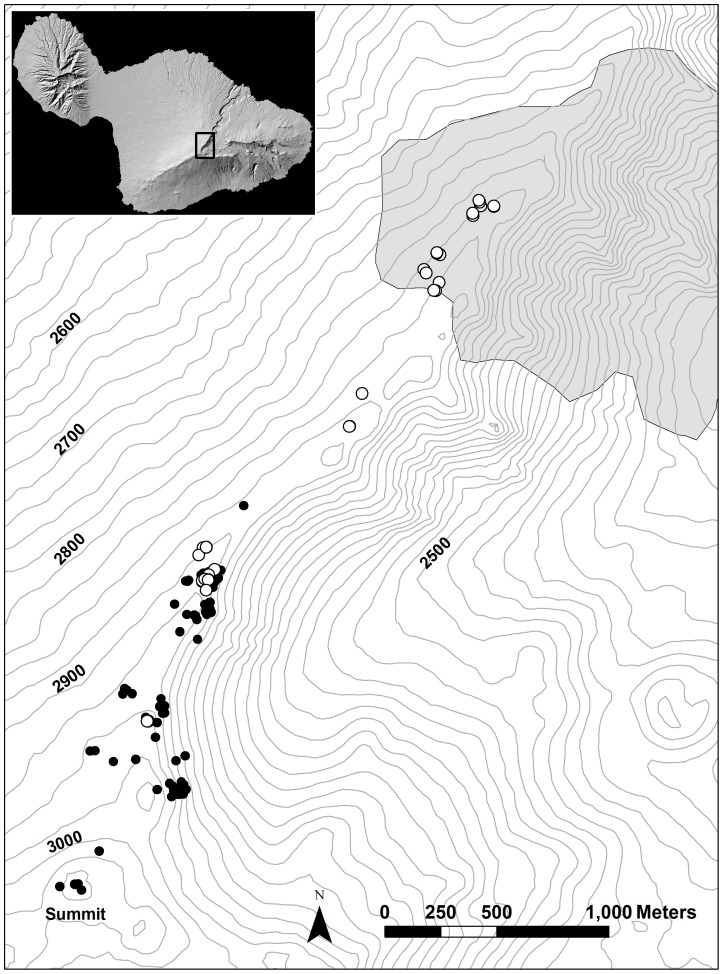
Map of study site. Black circles are locations of the 121 flowering plants along the western crater rim in 2007; white circles are locations of the 32 study plants. Many additional silverswords occur within the crater, to the east of the western rim plants (not shown). Argentine ant distribution indicated as gray polygon. Topographic lines are 25 m elevation contours. Small black rectangle in inset of Maui shows location of study site.

Invasive Argentine ants can form large, continuous populations of inter-connected colonies (supercolonies). At Haleakalā, two such populations are currently invading the park [46], however Argentine ants as yet overlap silverswords in only a small portion of the silversword range, along the western rim of the crater (Fig. 1). In the summer of 2007, a total of 121 plants flowered along the west rim up to the summit, a large number for this area. This west rim area formed the total study area, ranging from 2820 to 2955 m elevation and spanning a distance of 2925 m. All of the flowering plants were mapped using GPS and placed into one of three phenological categories based on their flowering status as of June 27: early bloomers had at least 50% of their capitula open (in anthesis); middle bloomers had less than 50% of their capitula open but the capitulescense stalk was already well developed; late bloomers had capitulescense stalks that were beginning to bolt but had no open capitula. These three categories had a roughly normal frequency distribution, with most plants in the middle bloomer category. Sixteen of the 121 flowering plants on the west rim occurred within Argentine ant invaded habitat, all at the northern (lower elevation) end of the study site (Fig. 1).

To assess the potential effects of ants on silversword reproductive ecology, I selected all 16 of these ‘invaded’ plants, plus an additional 16 ‘uninvaded’ flowering plants located along the remainder of the west rim in areas not yet invaded by ants (Fig. 1). I selected the 16 uninvaded plants such that they would represent a wide range in their degree of isolation from other flowering individuals, and that they would collectively encompass the range of isolation observed in the 16 invaded plants. No other criteria were used when selecting the study plants; for example, study plants were chosen haphazardly when aggregations of flowering plants presented multiple options of similarly isolated individuals. Subsequently, nearest flowering neighbor distances for each of the 32 study plants were measured with a tape measure if less than 20 m, and were measured in ArcGIS if greater than 20 m; this distance considered all 121 of the flowering individuals at the site. I also quantified a second measure of isolation, the number of plants flowering together in a cluster. Relative to nearest flowering neighbor distance, this metric may better indicate potential attraction of pollinators to a general area [8], [47], through aggregate visual or olfactory cues (silversword inflorescences are strongly aromatic). I assigned each study plant to flowering clusters, defined here as groups of flowering plants whose members were less than 50 m apart, and that were separated from other individuals or groups of flowering plants by at least 50 m (again considering all 121 flowering plants). This distance, although somewhat arbitrary, most effectively segregated the plants in the study area into clusters of different sizes. In addition, while it is unknown how far Hawaiian *Hylaeus* bees are capable of foraging, this distance scales reasonably with several anecdotal observations of bees typically remaining near their nests or localized clusters of plants [25], [48].

To characterize size of floral display in analyses, the total number of capitula was counted for each plant, and this number was multiplied by the average number of achenes among the three capitula collected for seed set analyses (see below), to obtain an estimate of the total number of florets per plant. This metric (log transformed) was strongly correlated with other measures of floral display size, including log(total number of capitula in inflorescence) (r = 0.905, p<0.001) and inflorescence height (r = 0.807, p<0.001). Although this estimate of floral display size overestimates the number of florets that are actually in anthesis at any given time, it was assumed that the proportion of open florets was similar among plants in a given phenology category, and that this overestimation should therefore not lead to serious biases when comparing plants on a relative scale.

Because silverswords on the crater’s west rim are arranged in a roughly linear fashion, the study plants were also located in a relatively straight line, forming a natural transect 2925 m long (Fig. 1). Because Argentine ants are currently located only on one end of this transect, it is possible that ant presence is confounded with other environmental characteristics that may vary systematically along the crater rim. For example, there is a moderate elevational gradient along the transect, with invaded plants situated at a mean elevation (±SE) of 2827±3.4 m and uninvaded plants situated at a mean elevation of 2905±6.8 m. This and/or other unknown factors (e.g. rainfall) could influence pollinator abundances or behavior in addition to, or instead of, ant presence. To test for this possibility, position along the transect was measured for each study plant in ArcGIS, and was incorporated in spatial analyses (see below).

Other flowering plant species could also influence pollinator behavior at silverswords. However, vegetation at the site is sparse, and the two most common species have flowering peaks that have only partial overlap with silverswords. I thus did not consider this to be an important confounding variable.

### Floral Visitation

I made a total of four 10-minute observations at each of the 32 study plants between July 6 and July 23, 2007. The four observations at each plant were distributed throughout the day: one each between 9∶30 and 11∶30 am, 11∶30 am and 1∶30 pm, 1∶30 and 3∶30 pm, and one dusk/night observation between 7∶45 and 10∶30 pm. On each observation day, approximately half of the observations were conducted on plants in the ant-invaded area and half on plants in the uninvaded area. I also altered the sequence of plant observation within each area on different days. During each timed observation, I counted the total number of unique floral visitors arriving on all of the capitula on one half of the capitulescence; I chose the half most directly facing the sun. I counted all insects that were strong fliers that actively moved between flowers on a given plant or between plants. Mostly sedentary insects, consisting mainly of *Nysius* seedbugs (Hemiptera: Lygaeidae), were not counted. To ensure that each visitor was counted only once in each 10-min observation period, I captured each visitor with a plastic snap-cap vial. To account for visitors that evaded capture, the captured visitor totals were augmented with the maximum number of uncaptured visitors (of each taxon) simultaneously seen on the capitulescence; because these may have sometimes represented different individuals during the course of the 10-minute time period, this was a minimum visitor count. At the end of each observation period, most captured floral visitors were released. Representatives of insects that could not be identified in the field, however, were retained for later identification (*Hylaeus* spp. were lumped together for these counts, so that identification to the species level was not attempted).

To assess whether floral visitors could be transferring pollen between flowers, a total of 119 visitors were retained, with captures biased towards the more common visitor taxa, and pollen grains on the external surfaces of these individuals were counted under a dissecting microscope. Several honeybee (*Apis mellifera*, Hymenoptera: Apidae) visitors carried large balls of pollen on their corbiculae; these pollen balls are densely packed and may be largely unavailable for pollination. For these individuals, two counts were performed: one excluding the pollen balls, and a second including the loads on the corbiculae. For the latter, the large pollen balls were subsampled to estimate the total load: a fraction of the balls were broken apart to count individual grains, and these counts were multiplied by the estimated relative size of the fraction. For a subset of specimens, all pollen was removed, stained with basic fuschsin and mounted in glycerin jelly [49]. The latter was done for the following taxa (n = number of specimens): *H. nivicola* (n = 19), *H. difficilis* (n = 2), *A. mellifera* (n = 1), *N. nubicola* (n = 11), *N. molokaiensis* (n = 2), *T. cratericola* (n = 3), *A. exotica* (n = 1), *E. tenax* (n = 1), *D. asketostoma* (n = 4), *S. latitergum* (n = 7), *C. monticola* (n = 1), *G. longipulvilli* (n = 1), *R. rhabdotis* (n = 1), and unidentified noctuid moths (n = 5). Slide mounted pollen was then examined under a compound microscope and compared with reference pollen samples collected from flowering plants at the study site, as well as with images in Selling [50]. It proved difficult to differentiate with confidence silversword pollen from that of other Asteraceae plants at the site, including *Dubautia menziesii*, *Tetramalopium humile*, *Gnaphalium sandwicensium* and *Hypochaeris radicata*. All Asteraceae pollen was therefore lumped together.

Caterpillars of *R. rhabdotis* can destroy substantial numbers of florets in infested capitula and hence potentially influence attractiveness to pollinators. I estimated the degree of *Rhynchephestia* damage for each study plant by haphazardly selecting one capitulum in the top and bottom halves of each capitulescense, and scoring caterpillar presence or absence in those capitula plus the nine closest capitula surrounding them (n = 20 capitula for each plant). Degree of *Rhynchephestia* damage was calculated as the percentage of infested capitula per plant.

To assess relative ant densities in the vicinity of each of the invaded study plants, I placed a standard bait consisting of blended tuna and corn syrup on an index card at the base of each of the plants on three occasions. I counted the number of ants on the cards after one hour, and averaged the three counts for each plant.

Patterns in floral visitation among the different diurnal time periods were analyzed using a one-way ANOVA. Patterns in visitor numbers among the 32 study plants were analyzed with two general linear models, one using all floral visitors combined and one using *Hylaeus* spp. only. Numbers of floral visitors were pooled across the four observation periods for each plant for the response variables. The following continuous and categorical explanatory variables were included in both models: ant presence (yes, no), floral display size (total number of florets per plant), nearest flowering neighbor distance, number of flowering plants in cluster, phenology category (early, middle; a single late plant was shifted to the middle category), and *Rhynchephestia* damage rate. Nearest flowering neighbor distance, number of plants in cluster, and floral display size were log transformed to improve linearity with the responses. None of the explanatory variables had inter-correlation coefficients greater than 0.52 or variance inflation factors (VIF) greater than 1.5.

To assess whether some unidentified environmental gradient along the study site, rather than ant presence, may better account for *Hylaeus* visitor numbers, I constructed an alternative model that included all of the above explanatory variables but substituted transect position for ant presence; I did not include both variables in the same model because they were confounded with one another. To investigate further the relative strengths of the alternative explanatory variables, I regressed residual *Hylaeus* visitor numbers, after controlling for all effects except ant presence, against transect position and fitted trend lines to each side of the ant invasion boundary. I then inspected whether a break in the overall trend occurred at the invasion boundary, as would be expected if visitor numbers are affected mainly by ant presence. In contrast, if an environmental gradient along the transect is the main determinant of *Hylaeus* numbers, there should be little if any break in the trend on either side of the boundary.

Because pollen load was not assessed on floral visitors in a standardized fashion, only general patterns are presented and statistical analyses were not performed on these data.

### *Hylaeus* Numbers at Pan Traps

To obtain an independent measure of the spatial abundance of the main native pollinator across the west rim study site, I used yellow pan traps to capture *Hylaeus* bees during the summer of 2009, a year in which there were no flowering silverswords on the west rim. This approach removes the potential influence of the locations of flowering silverswords on estimated spatial patterns of bees: silversword inflorescences represent large and showy resource patches that could partly obscure typical bee foraging and/or nesting spatial patterns by attracting them from a greater distance than most resources. I placed one pan trap every 75 m along the transect spanning the study site, from 0 to 2925 m, for a total of 40 pan traps. Pan traps consisted of yellow plastic bowls placed on the ground and filled with soapy water, and were opened for 1–2 days at a time, for a total of 4 days each, between August 9 and August 24, 2009. I also recorded whether a flowering individual of the shrub *Dubautia menziesii*, a common relative of the silversword with small yellow flowers, was present within 5 m of each pan trap. Number of *Hylaeus* captures per day was calculated for each trap, and used as the response variable in a two-way ANOVA, with ant presence (yes, no) and flowering *Dubautia* presence (yes, no) as the two fixed factors. As with numbers of *Hylaeus* visitors to silverswords, I assessed whether transect position better accounted for *Hylaeus* pan trap captures than did ant presence. I did this by constructing an alternate model with both transect position and flowering *Dubautia* presence included as explanatory variables. I also explored the pattern in *Hylaeus* captures graphically, by regressing residual *Hylaeus* captures, after controlling for the effect of *Dubautia* presence, against transect position and fitting trend lines to each side of the ant invasion boundary.

### Seed Set and Feeding Damage

From each of the 32 study plants, I collected three capitula between August 22 and August 25 after the florets had dropped and seeds had set, but before achenes began dropping from the capitulae [51]. For two late-blooming plants, the capitula were collected on October 1, 2007. At each plant, one capitulum was haphazardly collected from each of the top, middle and bottom third of the capitulescence.

The achenes from each capitulum were soaked in water and dissected under a microscope to determine if they possessed an intact, filled embryo and thus had been fertilized. At the same time, each achene was counted and scored for feeding damage. The two main herbivores on Haleakalā silversword inflorescences (*Rhynchephestia* caterpillars and *Trupanea* maggots) each produce characteristic and easily identifiable feeding damage. A third category, unknown herbivory, was used for damage usually characterized by small holes, perhaps produced by endemic *Nysius* seedbugs. The damage caused by *Trupanea* and especially *Rhynchephestia* can be extensive [35], and both herbivores typically initiate feeding before capitula and florets are open. Their effects on silversword reproduction can therefore stem from predation on viable seed embryos as well as prevention of seed set through the destruction of florets prior to or soon after fertilization.

I used general linear models to assess whether a variety of factors might be associated with degree of achene damage for individual plants. Achene damage was quantified as percentage of achenes per capitulum damaged by *Rhynchephestia*, *Trupanea*, or all sources of damage combined, and was averaged among the three capitula for each plant and log transformed as the response variable for each of the three models. Explanatory variables included in each model were ant presence, phenology category, nearest flowering neighbor distance, number of flowering plants in the cluster, and floral display size. Nearest flowering neighbor distance, number of plants in cluster, and floral display size were log transformed to improve linearity with the response variables. None of the explanatory variables had inter-correlation coefficients greater than 0.20 of VIFs greater than 1.1.

Seed set was calculated as the percentage of intact, filled achenes among all achenes (including damaged ones) per capitulum. Partially damaged achenes that had been fertilized, but whose filled embryos were reduced by herbivory (a rare occurrence), were excluded from the set seed total, as these seeds may not have been viable. Percent seed set was averaged among the three capitula for each plant, log transformed and used as the response variable in a general linear model. Explanatory variables included in the model were ant presence, phenology category, floral visitation rate (number of visitors per 100,000 florets per minute of observation), percent achene damage (from all sources of herbivory), nearest flowering neighbor distance, number of plants in the cluster, and floral display size. Visitation rate, percent achene damage, nearest flowering neighbor distance, number of plants in cluster, and floral display size were log transformed to improve linearity with the response variable. Because six of the study plants exhibited very low levels of seed set despite close proximity to another flowering plant (see Results), suggesting cross-incompatibility, I also repeated the above model using a reduced data set with those six plants removed to better assess the effects of spatial factors on percent seed set. None of the explanatory variables in either model have inter-correlation coefficients greater than 0.58 or VIFs greater than 1.8.

Fits of each explanatory variable with the response variable, leverage plots, and distributions of residuals were inspected to confirm the appropriateness of the general linear models used. Statistical analyses were performed in JMP 9.0.2 (SAS Institute Inc., Cary, NC, USA).

## Results

### Floral Visitation

**Figure 2:**
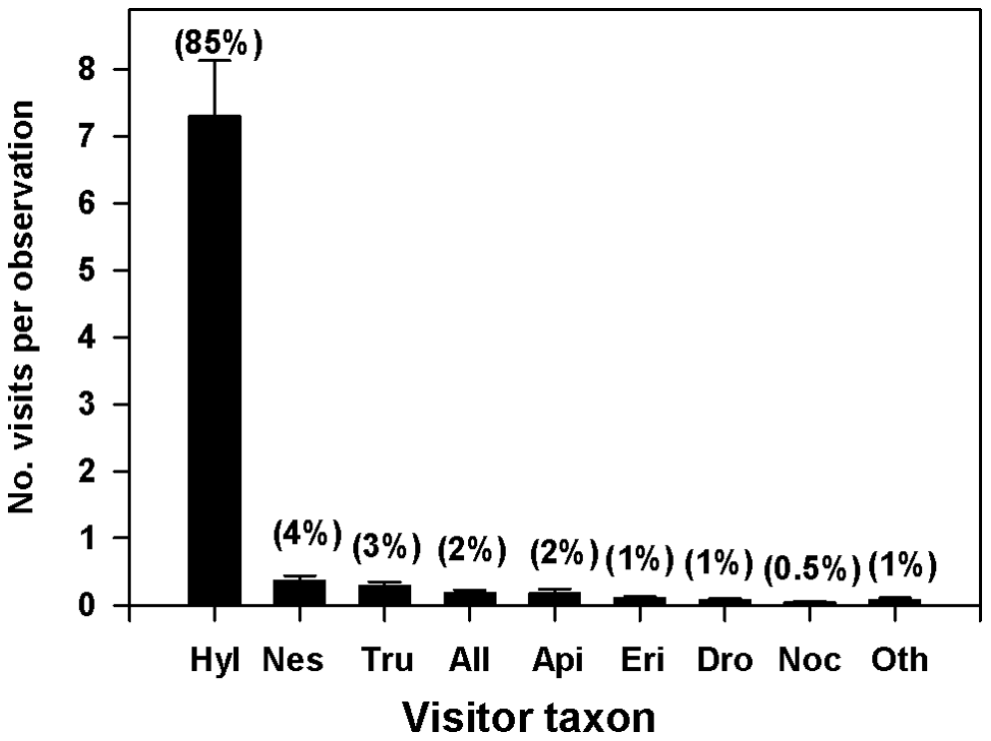
Mean number (±SE) of floral visits per 10 min observation of the most common visitors. Means are averages of all observations at all study plants. Percentages above bars indicate the proportion of total floral visits made by the visiting taxon. Abbreviations: Hyl = *Hylaeus* species, Nes = *Nesodynerus* species, Tru = *Trupanea* species, All = *Allograpta* species, Api = *Apis mellifera*, Eri = *Eristalis tenax*, Dro = Drosophilidae species, Noc = Noctuidae species, Oth = all other visitors.

At least 19 insect taxa were seen in a total of 1102 floral visits. Native *Hylaeus* species were by far the most common floral visitors, averaging 7.3 visits (±0.8 SE) per 10-minute observation period per plant and accounting for 85% of all visits observed (Fig. 2). Although most of these bees were not identified to species, of the 22 *Hylaeus* collected for pollen analysis, 19 (86%) were *Hylaeus nivicola* and 3 (14%) were *Hylaeus difficilis*. No other floral visitor made more than 4% of observed visits, and all averaged fewer than 0.4 visits per observation period. The other visitors included generalist pollinators and floral visitors such as honeybees (*A. mellifera*), hover flies (Diptera: Syrphidae; *Allograpta exotica*, *Allograpta obliqua*, *Eristalis tenax*), tachinid flies (Diptera: Tachinidae; *Chaetogaedia monticola, Gonia longipulvilli*), pomace flies (Diptera: Drosophilidae; *Drosophila asketostoma, Scaptomyza latitergum*), and other unidentified flies (Diptera: Muscidae and Calliphoridae) and moths (Lepidoptera, mostly Noctuidae), as well as insect predators (Hymenoptera: Vespidae; *Nesodynerus nubicola*, *Nesodynerus molokaiensis*, *Vespula pensylvanica*) and seed predators and stem gallers (Diptera: Tephritidae; *Trupanea cratericola, Trupanea limpidapex*). Of the visitor species listed, only the *Hylaeus* bees, *Drosophila*, *Scaptomyza* and *Trupanea* flies, and *Nesodynerus* wasps are native. Argentine ants were never observed visiting flowers, and indeed were never observed anywhere on the inflorescence, likely owing to the abundant sticky secretions produced by glandular hairs that cover all vegetative parts of the inflorescence. Only three floral visitors were seen during a total of 320 minutes of dusk/night observations: two noctuid moths and one moth belonging to an unknown family. Diurnal visits were spread evenly among the daytime periods (9∶30–11∶30 am: mean visits per 10-min observation session = 12.72±2.11 SE; 11∶30 am–1∶30 pm: mean = 11.63±1.41 SE; 1∶30–3∶30 pm: mean = 10.00±1.66 SE), with no significant differences in visitation rates amongthe three periods (one-way ANOVA, F = 0.61, p = 0.546).

**Figure 3:**
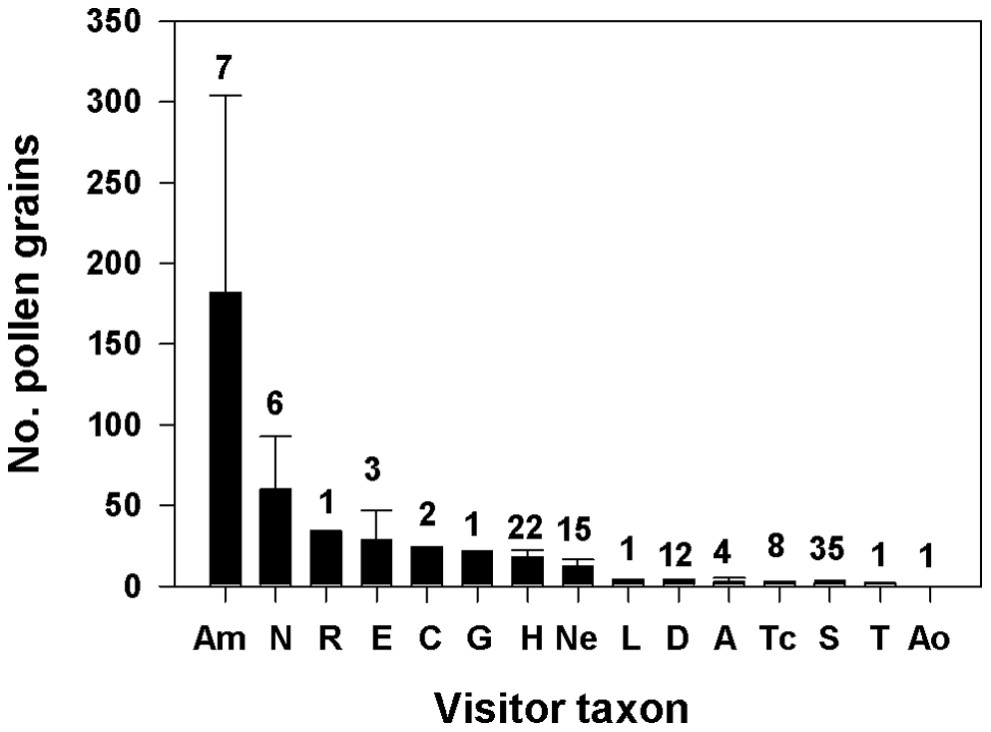
Mean number (±SE) of pollen grains externally attached to individuals of floral visitor taxa. Numbers above bars indicate numbers of individuals examined. Note break in y axis. Abbreviations: Am = *Apis mellifera* (does not include pollen balls on corbiculae), N = Noctuidae species, R = *Rhynchephestia rhabdotis*, E = *Eristalis tenax*, C = *Chaetogaedia monticola*, G = *Gonia longipulvilli*, H = *Hylaeus* species, Ne = *Nesodynerus* species, L = *Lispe* species, D = *Drosophila asketostoma*, A = *Allograpta exotica*, Tc = *Trupanea cratericola*, S = *Scaptomyza latitergum*, T = *Trupanea limpidapex*, Ao = *Allograpta obliqua*.

External pollen loads were higher on honeybees than all other taxa (Fig. 3; mean pollen grains on body not including pollen balls on corbiculae = 182±122 SE, n = 7; mean including pollen balls on corbiculae = 14,063 grains ±12,264 SE). In comparison, native *Hylaeus* averaged 18.0 (±4.2 SE, n = 22) pollen grains per bee. Noctuid moths had the highest pollen loads among all non-honeybee visitors (mean = 60.0 grains ±32.8 SE, n = 6), though sample sizes were very uneven among taxa. All specimens whose pollen was examined with a compound microscope were found to be carrying Asteraceae pollen, with the exception of one noctuid moth, which was carrying a single grain of what appeared to be *Leptecophylla tameiameiae* (Ericaceae) pollen. Several insects also carried a single grain of non-Asteraceae pollen in addition to their larger loads of Asteraceae pollen: *L. tameiameiae* on one of the *N. molokaiensis* individuals, and what appeared to be *Plantago lanceolata* (Polygonaceae) pollen on the other *N. molokaiensis* individual and on one *H. nivicola* individual.

Because *Hylaeus* comprised the vast majority of floral visitors, numbers of *Hylaeus* visitors were strongly correlated with numbers of total visitors (Pearson r = 0.990), and each of these responses yielded very similar results in general linear models assessing patterns of floral visitation (Table 1). Ant presence was significantly associated with visitor numbers after controlling for other significant factors, with plants in the invaded area receiving roughly half as many visitors as plants in the uninvaded area. Flowering phenology was strongly tied to visitor numbers, with early flowering plants receiving many fewer visitors than middle flowering plants. Also highly important in influencing visitor numbers in both models was a measure of plant isolation: as the number of flowering silverswords clustered within 50 m of each study plant increased, visitor numbers per plant decreased. Finally, floral display size significantly influenced visitor numbers, both among all visitors and *Hylaeus* only: plants with more florets attracted more visitors. In contrast, the nearest flowering neighbor distance and the degree of *Rhynchephestia* damage to the capitulescence had no significant influence on visitor numbers.

**Table 1:** Relationships between floral visitor numbers per plant and the explanatory variables considered.^1^

	All visitors				*Hylaeus* only			
Effect	Coefficient^2^	Fitted means^3^	F	p	Coefficient	Fitted means	F	p
ant presence	–	22.3±4.4a present	7.90	0.010	–	17.5±4.4a present	7.03	0.014
		40.2±4.3b absent				34.6±4.4b absent		
phenology category	–	18.3±4.9a early	15.49	0.001	–	13.5±5.0a early	13.99	0.001
		44.2±3.8b late				38.6±3.9b late		
log(dist nearest plant)	−2.88±4.05	–	0.51	0.484	−3.04±4.13	–	0.54	0.468
log(num plants in cluster)	−34.5±10.1	–	11.72	0.002	−33.3±10.3	–	10.53	0.003
log(num florets)	26.9±9.2	–	8.54	0.007	21.2±9.4	–	5.11	0.033
*Rhynchephestia* damage rate	−0.04±0.12	–	0.11	0.740	−0.02±0.12	–	0.04	0.839

1 Total model R^2^ = 0.64 for all visitors as response, and R^2^ = 0.59 for *Hylaeus* only as response.

2 Coefficients ± SE shown for continuous variables, least square fitted means ± SE shown for different levels of categorical variables.

3 Least square fitted means ± SE shown for different levels of categorical variables; levels within each categorical variable that are significantly different at α = 0.05 indicated with different letters, as determined by post hoc t tests.

**Figure 4:**
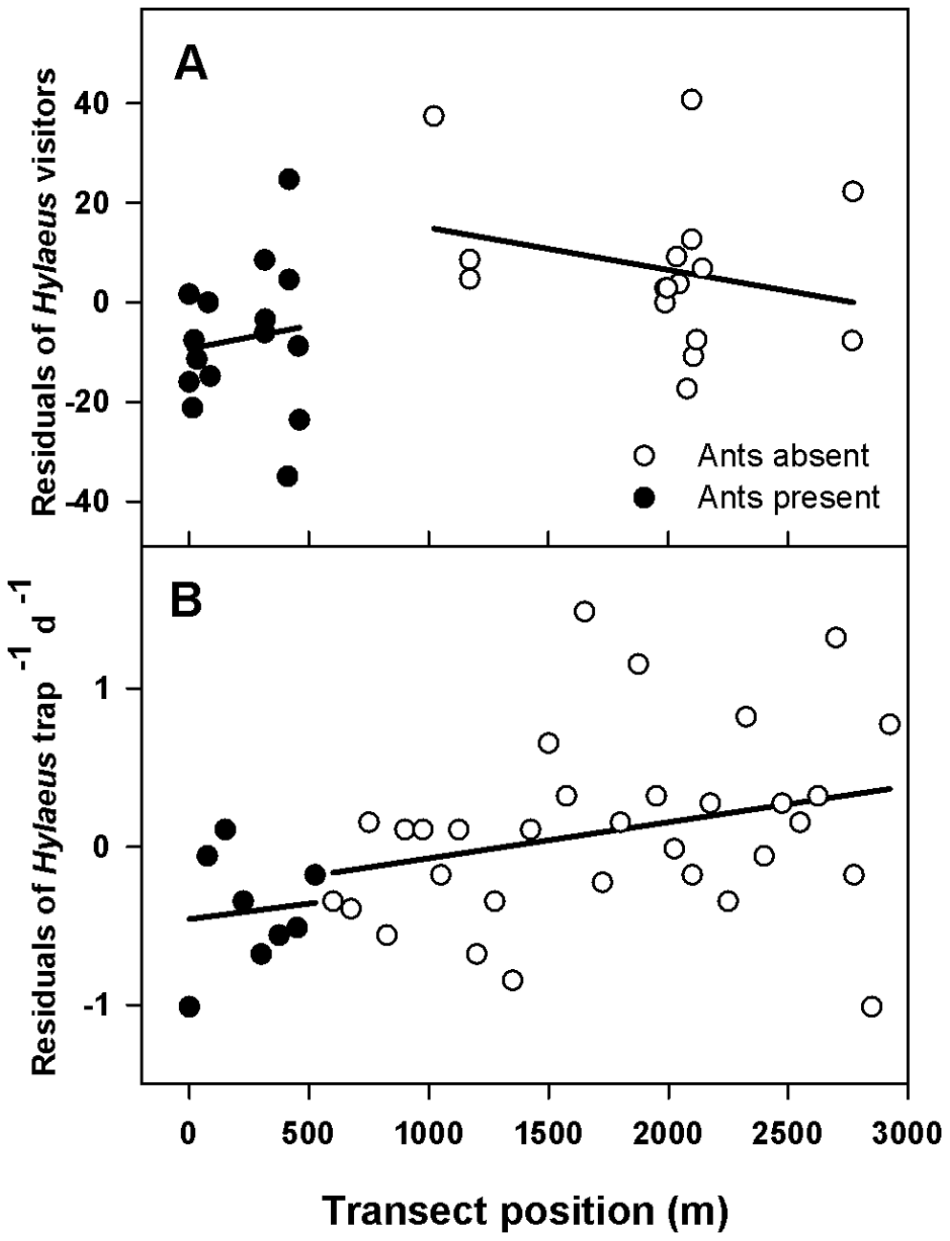
*Hylaeus* numbers as a function of location along the west rim study site. A) Residual numbers of *Hylaeus* visitors, after controlling for plant phenology and number of plants in cluster, to flowers at each of the 2007 study plants (summed over four observation periods), plotted against transect position. Linear regressions were fit to points on either side of the ant invasion boundary; neither slope is significantly different from 0 (n = 16, p = 0.844 for invaded area; n = 16, p = 0.239 for uninvaded area). B) Residual *Hylaeus* captures in yellow pan traps (per trap per day) in 2009, after controlling for presence of nearby flowering *Dubautia* plants, plotted against transect position. Slopes of regressions fit to points on either side of the invasion boundary are also not significantly different from 0 (n = 8, p = 0.815 for invaded area; n = 32, p = 0.125 for uninvaded area).

It is possible that other unmeasured environmental variables co-varying with ant presence may have actually been responsible for lower floral visitor numbers in the invaded area. When an alternative model was constructed, with the continuous variable ‘transect position’ substituted for the categorical variable ‘ant presence,’ transect position was significantly associated with *Hylaeus* visitor numbers (F = 4.64, df = 1, p = 0.042), with fewer *Hylaeus* at the end of the transect invaded by ants. However, transect position explained less of the variation in *Hylaeus* visitor numbers than did ant presence (adj. MS = 1332 and 1864 in each model, respectively). In addition, a break in the trend in *Hylaeus* numbers appears to exist at the ant invasion boundary (Fig. 4a). The regression trends on both sides of the boundary were not significantly different from 0 (Fig. 4a); however, even trends with slopes of 0 but offset from each other across the invasion boundary would suggest an abrupt shift in *Hylaeus* numbers, and would be generally inconsistent with a continuous environmental gradient along the transect being the main causal factor influencing *Hylaeus* numbers.

Within the invaded area, numbers of *Hylaeus* visitors were unrelated to ant densities around the study plants. A simple linear regression of *Hylaeus* visitors against log(mean ant density) was not significantly different from 0 (n = 16, t = −0.85, p = 0.409).

### Pan Traps

The two-way ANOVA model indicated that both ant presence and the presence of flowering *Dubautia* shrubs were associated with *Hylaeus* captures in yellow pan traps in 2009 (Table 2). Captures were higher near flowering *Dubautia* shrubs and lower in the ant-invaded area, but these two variables explained relatively little of the variation in *Hylaeus* captures (R^2^ = 0.24). When transect position was substituted for ant presence in a general linear model, it was significantly associated with *Hylaeus* captures in the pan traps (F = 8.42, df = 1, p = 0.006), with captures increasing as trap location along the west rim progressed from north to south, or upslope. Transect position explained more of the variation in *Hylaeus* captures than did ant presence (adj. MS = 2.40 *vs* 1.64 in each model, respectively). Finally, in contrast to the pattern obtained using 2007 *Hylaeus* floral visitation data (Fig. 4a), the 2009 *Hylaeus* pan trap captures show a much less obvious break in trend across the ant invasion boundary (Fig. 4b). Trends on either side of the invasion boundary were again not significantly different from 0 though, indicating that a stronger break would be present if flat slopes were fitted on either side of the ant boundary.

**Table 2:** Relationship between *Hylaeus* captures per day in yellow pan traps and the presence of Argentine ants and the proximate presence of flowering *Dubautia* shrubs.^1^

A. Effect	Fitted means^2^	F	p
ant presence	0.38±0.20a present	5.37	0.026
	0.89±0.10b absent		
flowering *Dubautia* presence	0.86±0.13a present	5.81	0.021
	0.41±0.16b absent		

1 Model R^2^ = 0.24.

2 Least square fitted means ± SE shown for different levels of categorical variables; levels within each categorical variable that are significantly different at α = 0.05 indicated with different letters, as determined by post hoc t test.

### Achene Damage

**Figure 5:**
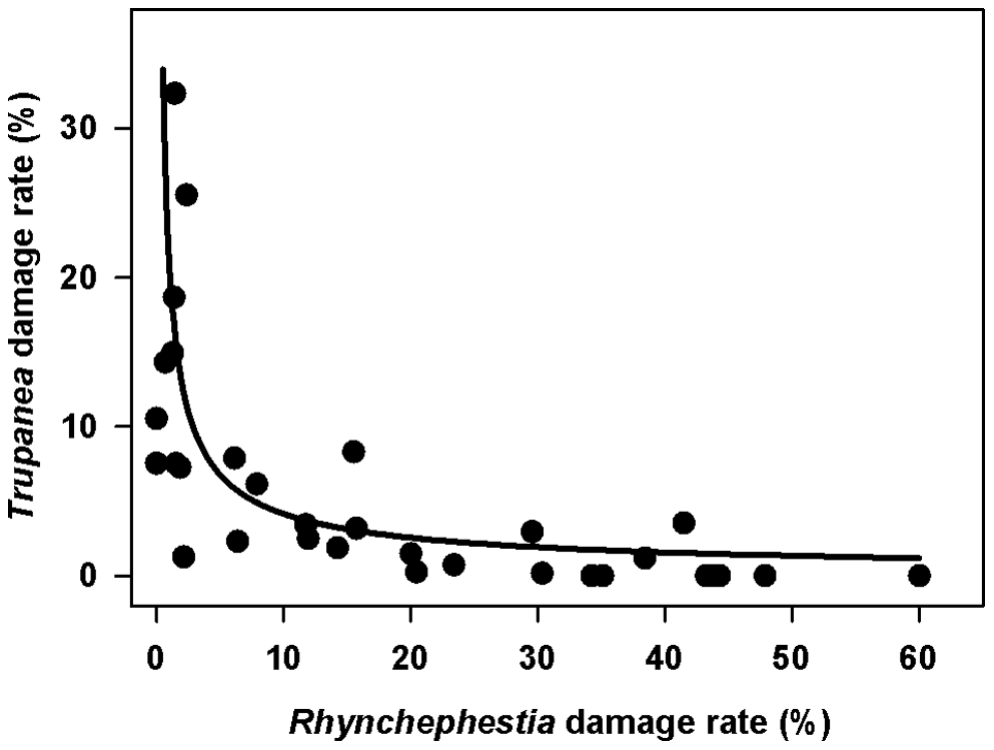
Relationship between *Rhynchephestia* damage rate and *Trupanea* damage rate, per plant. Fitted curve is back-transformed from the log-log relationship between the damage rates (log-log trend R^2^ = 0.67, p<0.001).

Achene damage ranged from 4.1 to 60.0% among individual plants, with a mean percentage of 25.4±2.5 SE of achenes damaged by some herbivore. This herbivory was dominated by *Rhynchephestia* caterpillars, with single individuals capable of chewing through substantial portions of the field of immature achenes and florets in a capitulum. *Rhynchephestia* damaged on average 19.2±3.1% of the achenes of the silversword study plants, representing an average of 57% of the total achene damage per plant. *Trupanea* larvae are much smaller than *Rhynchephestia* larvae, and each pupa found was associated with an average of 4.3 damaged achenes surrounding it. The number of *Trupanea* pupae found per capitulum ranged from 0 to 23, and averaged 3.7. *Trupanea* damage per plant averaged 5.8±1.4% of achenes, representing on average 36% of the total achene damage per plant. Log transformed percent damage by each herbivore, per plant, was strongly negatively correlated with that of the other (Pearson r = −0.82, n = 32, p<0.001); plants with more than about 5% damage from one herbivore had very low damage from the other herbivore (Figure 5).

**Figure 6:**
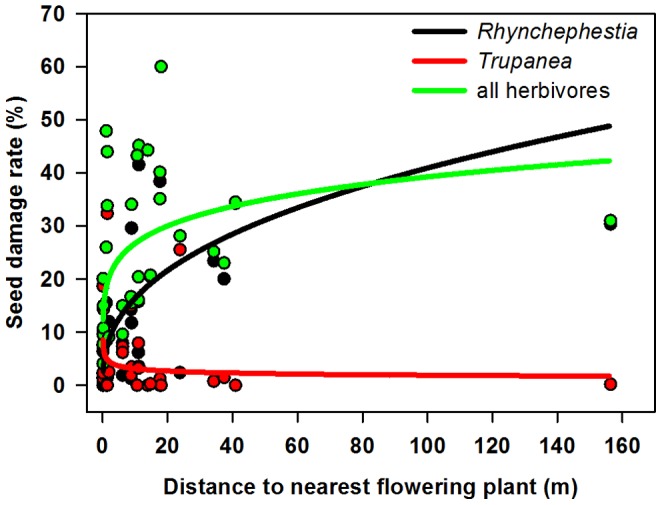
Seed damage rates as a function of plant isolation. Isolation measured as distance to the nearest flowering plant. Fitted curves are back-transformed from the log-log relationships for each class of herbivore (for *Rhynchephestia*, log-log trend R^2^ = 0.42, p<0.001; for *Trupanea*, log-log trend R^2^ = 0.18, p = 0.016; for all herbivores, log-log trend R^2^ = 0.34, p = 0.001).

The general linear model indicated that *Rhynchephestia* damage was significantly associated with only one of the explanatory variables considered, the nearest flowering neighbor distance, with percent damage increasing as this distance increased (Table 3). The back-transformed fitted relationship with this single significant explanatory variable is shown in Figure 6. *Trupanea* damage was also significantly associated only with nearest flowering neighbor distance, but in this case percent damage decreased as this distance increased (Table 3, Fig. 6). Because *Rhynchephestia* was the dominant herbivore in silversword capitula, the general linear model for total percent achene damage was very similar to the model for *Rhynchephestia* damage. Only a positive association between nearest flowering neighbor distance and total achene damage was evident (Table 3, Fig. 6).

**Table 3:** Relationships between log-transformed achene damage rates per plant (classified by source of herbivory) and the explanatory variables considered.^1.^

	*Rhynchephestia*		*Trupanea*		All damage	
Effect	F	p	F	p	F	p
ant presence	0.15	0.699	0.37	0.547	0.22	0.643
phenology category	0.22	0.642	0.07	0.790	0.29	0.598
log(dist nearest plant)^2^	14.20	0.001	5.37	0.029	10.14	0.004
log(num plants in cluster)	1.88	0.182	0.00	0.970	1.73	0.200
log(num florets)	0.50	0.484	1.91	0.179	0.88	0.358

1 Total model R^2^ = 0.49 for *Rhynchephestia* damage as response, R^2^ = 0.25 for *Trupanea* damage as response, and R^2^ = 0.45 for all damage as response.

2 Coefficient of log(dist nearest plant) = 0.37±0.10 SE for log(*Rhynchephestia* damage rate),

= −0.23±0.10 for log(*Trupanea* damage rate), = 0.15±0.05 SE for log(all damage rate).

### Seed Set

Seed set per plant ranged from 0 to 55.4%, with a mean of 7.4±2.1% SE. The general linear model indicated that only the log nearest flowering neighbor distance was clearly significantly associated with the log percent seed set of the study plants (Table 4), with seed set decreasing as plant isolation increased. Floral display size, as measured by the log total number of florets, had a marginally significant positive association with log percent seed set (Table 4). None of the other spatial or plant-specific factors, including ant presence, floral visitation rate, percent seed damage, phenology category, or number of flowering plants in the cluster, appeared to influence a plant’s seed set. Because the effect of ants on seed set would presumably result from their impact on floral visitation, including both of these factors in the model might obscure an ant effect. However, when log visitation rate was removed from the model, ant presence was still not significantly associated with log percent seed set (F = 0.07, p = 0.801). In fact, the fitted least squares means of log percent seed set for plants in the invaded and uninvaded areas were nearly identical (0.69±0.11 SE and 0.68±0.10 SE, respectively).

**Table 4:** Relationships between log-transformed seed set rates per plant (for full and reduced datasets, see text) and the explanatory variables considered.^1.^

	Full dataset		Reduced dataset	
Effect	F	p	F	p
ant presence	0.01	0.937	0.32	0.579
phenology category	0.00	0.953	0.70	0.413
log(dist nearest plant)^2^	7.95	0.009	38.91	<0.001
log(num plants in cluster)	1.35	0.257	0.34	0.566
log(num florets)^3^	3.27	0.083	0.05	0.818
log(visitation rate)	0.47	0.499	0.82	0.378
log(seed damage rate)	2.15	0.155	0.72	0.408

1 Total model R^2^ = 0.39 for full dataset, and R^2^ = 0.77 for reduced dataset.

2 Coefficient of log(dist nearest plant) = −0.30±0.10 SE for full dataset, = −0.58±0.09 for reduced dataset.

3 Coefficient of log(number of florets) = 0.43±0.24 SE for full dataset.

**Figure 7:**
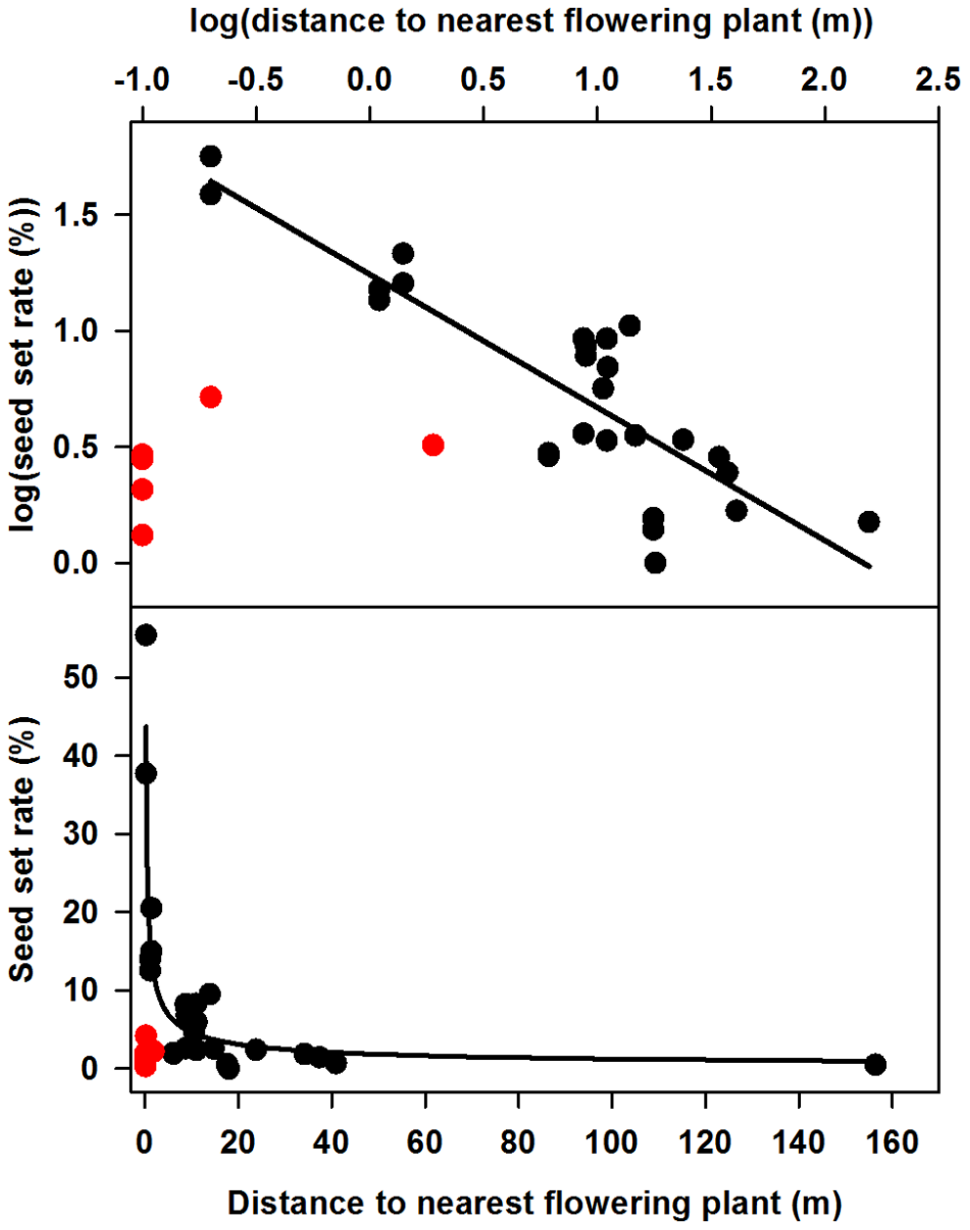
Seed set rates as a function of plant isolation. Isolation measured as distance to the nearest flowering plant. Top panel shows the log-log relationship (trend R^2^ = 0.73, p<0.001), bottom panel shows the back-transformed fitted curve of the same relationship. Red points are the six study plants hypothesized to be located adjacent to an incompatible neighbor; these points were excluded when fitting the curves in the panels.

The above full model explained relatively little of the variation in log percent seed set (R^2^ = 0.39). Part of this poor fit appeared to result from cross-incompatibility between six study plants and their nearest flowering neighbors, which would partly obscure the spatial effect of plant isolation on seed set rate: the plants in each pair were separated by 0.1, 0.2 and 1.9 m, yet five of the six plants had seed set rates of less than 2.5%, and the sixth had a seed set rate of just over 4%. When these six plants were removed from the dataset, the regression model explained much more of the variation in log percent seed set (R^2^ = 0.77) and suggested an overwhelming role of plant isolation, with log distance to nearest flowering plant being highly significant and no other factors being close to significant (Table 4). The nearest flowering neighbor distance alone explained 73% of the variation in seed set in this reduced dataset (Fig. 7).

## Discussion

### Floral Visitation

At least 19 insect taxa, in multiple orders, were observed visiting silversword flowers, which could suggest a relatively generalized pollination system [52], [53]. However, not all floral visitors are equally important [54], as some may be ineffective pollinators, obtaining floral rewards without reliably transferring pollen, or may visit only infrequently [9], [12], [55]. Pollinator importance is therefore typically evaluated as a combination of visitation rate and effectiveness per visit, and can reveal large differences among a suite of floral visitors [56]. For Haleakalā silverswords, endemic *Hylaeus* bees were the overwhelmingly dominant taxon in terms of visitation rate. In addition, all individuals examined carried external pollen morphologically consistent with silversword pollen. The inference that most of this was indeed silversword pollen, as opposed to that of other Asteraceae species at the site, is supported by the results of Wilson et al. [57], who sequenced DNA from the pollen carried in the internal crops of the same *Hylaeus* individuals captured and examined for external pollen in the present study: of 14 individuals that possessed pollen in their crops, 12 had ingested silversword pollen exclusively.

On the other hand, *Hylaeus* bees carry most of the pollen they collect internally and are not very hairy [34], and thus had relatively few pollen grains externally attached and available for pollination (Fig. 3). In comparison, introduced honeybees had much higher external pollen loads. This discrepancy in pollen loads could influence the relative effectiveness, and hence importance, of these two taxa as silversword pollinators [58]–[60]. I did not attempt to measure pollinator effectiveness, but several recent reviews suggest that frequency of visitation usually contributes much more to pollinator importance than does effectiveness per visit [61], [62]. If also true in this case, it would indicate that, at this site, *Hylaeus* bees are currently of predominant importance for Haleakalā silversword reproduction, and that, unless other major pollinators have now declined or disappeared (see below), silverswords may be fairly specialized in their pollination relationship with *Hylaeus*. This supports other assessments that have highlighted the central role of *Hylaeus* species in the pollination of native Hawaiian plants [34]–[36], [48], [63], [64], and points to the urgency of conserving this imperiled group of endemic bees [63].

Several factors appeared to significantly influence floral visitation (Table 1). The importance of one plant-specific characteristic, flowering phenology, was almost certainly an artifact of the timing of my study. I began observations after early flowering plants were past their peak, with many of their florets having already senesced, and this was the likely reason that these plants had many fewer visitors. After controlling for flowering phenology, I found that larger plants with larger floral displays attracted more visitors than did smaller plants with smaller displays, as in a number of other plant-pollinator systems [8], [47], [65], [66]. In contrast, numbers of floral visitors were unrelated to the nearest flowering neighbor distance, and I found that the number of additional flowering plants clustered nearby (<50 m) was negatively associated with numbers of *Hylaeus* visitors and total visitors. Other studies have typically found that floral visitor numbers decline with increasing isolation of plants or plant patches, or with decreasing size or plant density of patches [15], [18], [22], [47], [67], [68], including an example with *Hylaeus* in Hawai’i [48]. These patterns seem opposite to the one I observed. However, the studies cited typically examined the effects of increasing isolation or decreasing size of habitat fragments (separated by areas less suitable to pollinators), whereas in my case individual flowering silverswords differed in their degree of isolation but within a landscape that is unfragmented with respect to disturbance, plant invasion, or other general attributes of habitat quality. If other, more spatiotemporally regular plant resources determine local densities of *Hylaeus* at this site, and if *Hylaeus* forage relatively short distances from their burrows, then the pattern I observed would naturally emerge, as a set total of foraging bees inhabiting a given area would necessarily be divided among the number of flowering silverswords in that area (see [17] for a similar pattern).

My finding that the presence of invasive Argentine ants was associated with significantly lower numbers of silversword floral visitors, including *Hylaeus* bees, is congruent with previous findings of lower densities of *Hylaeus* nests in Argentine ant invaded areas at Haleakalā [25]. Predation and/or competitive exclusion of adult and immature bees at terrestrial nesting or resting sites could lead to decreased rates of floral visitation even if, as was the case here, no interference occurs at flowers (see [69]–[71] for examples of pollinator suppression through predation). The inference that ants were the causal factor behind the roughly 50% lower abundance of *Hylaeus* visitors in the invaded area is supported by the fact that plant invaded status was a better predictor of visitor numbers than was plant position along the linear study site, and by the break in the spatial trend in visitor numbers across the invasion boundary (Fig. 4a). However, spatial patterns in numbers of bees captured in yellow pan traps at the study site provided some evidence that an unidentified environmental gradient may affect *Hylaeus* abundances instead of, or in addition to, the presence of Argentine ants (Table 2, Fig. 4b). The evidence that Argentine ants were responsible for the lower numbers of *Hylaeus* visitors to silversword flowers at the northern, invaded end of the study site therefore remains equivocal.

### Herbivory in Flower Heads

In addition to providing nectar and pollen to floral visitors, silverswords represent an important resource for endemic seed predators. Indeed, for *R. rhabdotis*, no other host plant has been recorded [72]. Extensive damage of flower heads by these insects had long been recognized [37], [38], but Kobayashi [35] was the only previous worker to quantify it. Kobayashi estimated achene damage of up to 90% in some locations in 1971, with an overall average of 60% among 12 sampled areas within Haleakalā crater [35]. In comparison, I observed achene damage for individual plants that ranged from 4.1 to 60.0%, and averaging 25.4%, at the western crater rim site. My lower figures could be due in part to differences in methodology, or to differences in herbivore abundances between my higher elevation site and Kobayashi’s lower elevation sites, between different years, or both. Seed predation in Asteraceae plants by tephritid flies and Lepidoptera is a widespread phenomenon, and a prior study reported differences in predation rates across elevation [39].

I found that *Rhynchephestia* caterpillars were responsible for much more pre-dispersal achene damage than were *Trupanea* fruit fly larvae, and that there was a strong negative association between the two types of damage at the plant level (Fig. 5). Spatially, this partitioning of flowering individuals manifested as significantly increasing achene damage by *Rhynchephestia* with increasing nearest flowering neighbor distance, and an opposite trend for damage caused by *Trupanea* (Table 3, Fig. 6). The reason for the positive bias towards isolated plants on the part of *Rhynchephestia* is unknown, but similar patterns have been documented for other plants and their pre-dispersal seed predators [22]–[24]. One potential explanation involves predator satiation, whereby seed predator densities per host plant decline as plant densities increase, particularly in cases where ovipositing adults are strong dispersers, host plant detectability is high, and small or isolated plant populations consequently attract similar numbers of herbivores as larger or less isolated populations [22], [23]. Another mechanism may involve behavioral responses in which adult moths increase oviposition rates on isolated plants or at population edges [24], [73]–[75]. Such behavior may be adaptive when host plants are normally sparsely distributed [74], which is the case for Haleakalā silverswords: flowering individuals typically comprise a small fraction of the total population, and their distributions vary greatly both regionally and from year to year. The observed pattern could also result, in part, from lower rates of parasitism of caterpillars on isolated plants [24], [76].

The opposing spatial pattern for *Trupanea* abundance and feeding damage may indicate a bias towards selecting more aggregated host plants (as predicted by the resource concentration hypothesis, reviewed in [77]), or perhaps more likely, may indicate negative interactions between the two herbivores. Given the higher prevalence and more extensive feeding of *Rhynchephestia*, it seems likely that the strong spatial partitioning would be driven mainly by avoidance of *Rhynchephestia*-infested plants by *Trupanea* females, or by the active destruction of *Trupanea* larvae via nonselective feeding by *Rhynchephestia* caterpillars.

Patterns of achene damage resulting from all types of herbivory were very similar to those caused by *Rhynchephestia* alone (Table 3, Fig. 6), because of the strong effect of this species. Thus, spatial isolation, as measured by distance to the nearest flowering neighbor, appeared to be the sole factor under consideration that influenced overall levels of achene damage for plants at this site. Another measure of isolation, the number of flowering silverswords clustered nearby, was not significantly associated with degree of feeding damage, nor were the plant-specific attributes floral display size and flowering phenology. Argentine ants had no significant effect on levels of herbivory, likely owing to their absence from silversword inflorescences.

### Seed Production

I observed a wide range in estimated seed set per plant (0–55%), indicating vast differences in viable seed production. Seed set, as defined here, is the net outcome of the two largely opposing processes discussed above: cross-pollination and seed predation. Despite the potential for complex dynamics within and between these two processes, I observed only a relatively simple spatial pattern in seed production: only the nearest flowering neighbor distance had a clear significant association with a plant’s percent seed set, with more isolated plants setting many fewer seeds (Table 4). Very closely spaced plants also sometimes exhibited very low seed set, most likely as a result of cross-incompatibility. I did not directly test cross-compatibility, but a study of the Mauna Kea silversword indicated that half- and full-siblings are often cross-incompatible (e.g., <1% seed set, equivalent to selfing rate), as would be expected under the presumed sporophytic incompatibility system [44]. Similarly, Carr et al. [41] found that seed set in Haleakalā silverswords was occasionally unusually low (<1%) when hand-crossed with nearby plants, and hypothesized that this resulted from close relatedness and concomitant incompatibility between those individuals. Silversword seeds have generally poor dispersal abilities, with the vast majority falling less than 1 m from the maternal plant [51], often resulting in highly clumped seedling recruitment. This is likely to produced a pattern of siblings often growing in close proximity to one another, some of which will share self-incompatibility alleles.

A degree of cross-incompatibility among clustered plants may be a significant feature of the silversword breeding system and life history, depending on the degree to which siblings develop and flower in synchrony. Even so, the central importance of plant isolation on outcross-pollen transfer and hence seed set was clearly indicated when I excluded six plants from the dataset (Table 4, Fig. 7), on the grounds that each of these appeared to be flowering immediately adjacent to an incompatible individual. If this assumption is correct, it indicates that at this site, seed set dropped below 5% on average as the nearest compatible neighbor distance exceeded about 10 m (Fig. 7). A decline in seed production with increasing isolation matches my expectations and many previous findings [7], [8], [18], [19], [22], [48], including those for another long-lived monocarpic alpine rosette plant (*Frasera speciosa* [78]), but this is nonetheless an extremely steep gradient.

The percent seed set in some highly aggregated plants (e.g. 20–55%) was similar to that obtained in hand-crossed pollen augmentation experiments for silverswords (mostly 21–52%, but up to 84% seed set [36], [41], [44]), indicating that closely spaced flowering plants at my site were generally not pollen limited. In contrast, plants separated from flowering neighbors by even relatively short distances showed strong evidence of pollen limitation. This pollen limitation resulted not from reduced floral visitation, but instead from a failure of floral visitors to transfer non-self pollen across these distances; plants isolated by more than about 40 m exhibited levels of seed set consistent with self-pollination [36], [41]. It may thus be inferred that the presumptive main pollinators at this site, *Hylaeus* bees, tend not to visit multiple flowering plants during individual foraging bouts unless those plants are very close to one another. Prior studies have documented a greater tendency for pollinators to visit more flowers per plant before moving on to other conspecific individuals when distances between them are greater [7], [79], and this may be especially likely for silverswords. Silversword inflorescences each contain many thousands of florets, and even though all florets do not present pollen or offer nectar simultaneously, each plant may nevertheless often supply much more pollen and/or nectar than individual bees can carry in their crops, reducing the need for sequential visits to multiple plants.

It is important to note, however, that the extreme gradient in percent seed set measured at this site may not be representative of pollination dynamics across the entire Haleakalā silversword range, or from year to year. A separate set of 15 flowering plants located at lower elevations within Haleakalā crater in 2009 exhibited a substantially weaker rate of decline in seed set with isolation distance, including seed set of over 16% for a plant located more than 120 m from its nearest flowering neighbor (P. Krushelnycky, unpub. data). In an analogous situation, Powell [44] documented a sharply declining relationship between percent seed set and nearest flowering neighbor distance for Mauna Kea silverswords, that was very similar to the one I report in the present study. She noted that floral visitors at that site were dominated by *Hylaeus* bees and flies, whereas plants at another site that received frequent floral visitation by moths had significantly higher seed set. She therefore hypothesized that, in her system, moths may be more effective at moving pollen between distant plants than bees and flies. Moths comprised only 0.5% of floral visitors in my study, but it is unknown whether this is unusually low for this site or for the Haleakalā population as a whole. Similarly, it is unknown whether *Hylaeus* bees may exhibit different densities and/or foraging behavior across the silversword range, in response to spatial variation in other floral resources, nesting habitat or climatic factors.

The relative unimportance of the other factors in influencing percent seed set is also of interest. Although rate of floral visitation would seem to be critical given the silversword’s reliance on pollinators, plants at this site generally experienced ample insect traffic at their flowers, provided that they were not past their peak. Hence, for closely spaced plants where cross-pollination was prevalent, the observed degree of variation in visitation rate was apparently not enough to significantly affect pollination rates. As a result, the factors found to influence floral visitor numbers, such as floral display size, number of additional flowering plants clustered nearby, and possibly ant presence, also had no significant effect on silversword seed production (Table 4). Evidently, even the roughly 50% lower rate of floral visitation in the Argentine ant invaded area did not translate into lower seed set for those plants.

Likewise, the sometimes dramatic damage caused by native floral herbivores, which long ago prompted recommendations to protect silversword inflorescences with insecticide and netting [38], appears to be relatively unimportant in influencing total seed production. As Kobayashi [35] pointed out, endemic insects should not normally be expected to threaten severely the persistence of their natural host plant, and my results support this view. Although achene damage was sometimes substantial, it was not significantly associated with percent seed set (Table 4). It is important to recognize that percent achene damage is not equivalent to percent seed damage, because even under the most favorable scenarios (e.g. out-cross pollen augmentation), a large proportion of silversword achenes fail to set seed, possibly due to resource limitation [36]. Moreover, in my study, achene damage tended to be higher among more isolated plants, where this damage made little difference because of the lack of effective cross-pollination. Among aggregated plants, floral head herbivory clearly reduced the production of viable seeds to some degree, but the effect of this was strongly outweighed by the substantial cross-pollination occurring at these plants.

Haleakalā silverswords may also escape high levels of seed predation via the strong inter-annual variation in flowering incidence, which resembles the mast seeding dynamics that increase seed survival via predator satiation in high-flowering years [80]. A similar dynamic has been reported for *F. speciosa* [78], [81]. Both spatial and temporal mechanisms may therefore lessen the impacts of native seed predators on silversword reproduction. It is also worth noting that the degree of *Rhynchephestia* damage did not appear to influence floral visitor numbers (Table 1), and that adults of both *Rhynchephestia* and *Trupanea* were occasionally observed visiting flowers (Figs. 2 and 3). Both species may therefore perform some pollination in addition to seed predation. If the foraging behavior of either species differs substantially from that of *Hylaeus*, for example by transferring pollen across larger distances, their pollination services may be more important than inferred from their low visitation rates.

## Conclusions

Silversword inflorescences and infructescences attracted a wide variety of insects, suggesting that this ephemeral source of nectar, pollen and seeds plays an important ecological role in the relatively barren alpine environment at the top of Haleakalā volcano. My results confirm the interacting importance of insect pollinators and plant spatial dynamics in silversword reproduction. Silverswords rely on insects to outcross, yet the effectiveness of this biotic interaction is strongly influenced by the distance that pollen must be transferred between flowering individuals. The resultant Allee effect will vary in intensity across space and time [36], but nevertheless highlights the importance that population size and fragmentation can have for successful reproduction. If Haleakalā silversword numbers continue to decline, as they have over recent decades [42], this will increasingly result in fewer synchronously flowering plants, which on average will be spaced further apart. Management actions that can minimize future fragmentation or isolation of silversword aggregations will benefit reproduction, and cross-pollination distances should be considered in outplanting efforts.

Similarly, threats that severely reduce pollinator numbers can be expected to impact silversword reproduction. Although I found no association between pollinator visitation rates and percent seed set, this resulted from the fact that floral visitors were abundant at the study site, and that variation in visitation rates was moderate. At some point, however, increasingly severe reductions in pollinator numbers will inevitably impact pollination rates. It therefore seems premature to dismiss the potential effects of invasive ants on silversword reproduction. Argentine ants have to date invaded only about 2% of silversword habitat, yet appear to be capable of invading virtually all areas supporting silverswords [46], [82]. If ants do indeed suppress *Hylaeus* and other pollinator numbers, an effect on pollination rates may become evident when exerted at larger scales. In addition, ant densities can vary spatially and fluctuate over time, which may explain in part the difference between my study and that of Cole et al. [25] in estimated impact on *Hylaeus* numbers.

Although my results suggest that the dominant floral visitors, *Hylaeus* bees, may typically cross-pollinate only plants that are close to one another, other less common floral visitors may move pollen across greater distances, if less frequently [10]. Conservation of the wide range of insects that visit silversword flowers may therefore be important for preserving normal patterns of gene flow. This goal is likely to apply to rare or threatened plants more broadly. The silversword system also illustrates how spatial dynamics may differentially influence patterns in pollination and seed predation by native insects, and how this may minimize reproductive limitation in their host plants.
